# Advancing proteomic discovery through optimized multi-stage scoring and deep learning-enhanced open search

**DOI:** 10.1093/bioinformatics/btag224

**Published:** 2026-07-07

**Authors:** Chen Qian, Kaifei Wang, Pengzhi Mao, Ranfei Chen, Hao Chi

**Affiliations:** Institute of Computing Technology, Laboratory of Intelligent Information Processing of Chinese Academy of Sciences (CAS), Beijing, 100190, China; University of Chinese Academy of Sciences, Beijing, 100190, China; Institute of Computing Technology, Laboratory of Intelligent Information Processing of Chinese Academy of Sciences (CAS), Beijing, 100190, China; University of Chinese Academy of Sciences, Beijing, 100190, China; Institute of Computing Technology, Laboratory of Intelligent Information Processing of Chinese Academy of Sciences (CAS), Beijing, 100190, China; University of Chinese Academy of Sciences, Beijing, 100190, China; Institute of Computing Technology, Laboratory of Intelligent Information Processing of Chinese Academy of Sciences (CAS), Beijing, 100190, China; University of Chinese Academy of Sciences, Beijing, 100190, China; Institute of Computing Technology, Laboratory of Intelligent Information Processing of Chinese Academy of Sciences (CAS), Beijing, 100190, China; University of Chinese Academy of Sciences, Beijing, 100190, China

## Abstract

**Motivation:**

Protein search engines are essential for interpreting mass spectrometry data into biological insight. Current tools often face limitations in sensitivity when analyzing complex modern datasets, and lack a unified framework that effectively integrates deep learning features for both restricted and open searches, especially for scenarios aimed at discovering unknown modifications.

**Results:**

We present pFind+, a high-performance search engine for data-dependent acquisition (DDA) proteomics, extending pFind. It introduces an enhanced raw scoring that delivers substantially improved pre-filtering ability, while recovering most of the computational overhead through a tailored acceleration strategy. Coupled with an enhanced rescoring framework that effectively integrates deep learning features, pFind+ uniquely supports high-sensitivity, DL-enhanced open search, enabling comprehensive PTM discovery while incorporating hardware-aware inference optimizations for practical deployment. Evaluations across diverse datasets demonstrate its superior sensitivity, with gains of 12.7%–29.3% (average 17.9%) in restricted search and 8.0%–38.4% (average 25.8%) in open search over the best existing tools.

## Introduction

Proteomics aims to systematically characterize proteins in biological samples, including identities, expression levels, and post-translational modifications (PTMs), thereby providing insights into biological mechanisms and disease biomarker discovery. Liquid chromatography coupled with tandem mass spectrometry (LC-MS/MS) has become the cornerstone of modern proteomics due to its high resolution and accuracy. In a typical workflow, proteins are digested into peptides, separated by chromatography, and fragmented to produce MS/MS spectra. Protein search engines function as the essential computational tools that translate these spectral data into biologically meaningful information. Their performance directly governs the analytical depth and reliability.

Database searching represents the primary strategy for interpreting MS/MS data, with its performance critically dependent on the design of the scoring function. In the restricted search paradigm, tools such as pFind (Wang et al. 2007), MaxQuant (Cox and Mann 2008), Comet (Eng et al. 2013), and MSFragger (Kong et al. 2017) operate with constrained modification lists, employing specialized scoring algorithms to achieve efficient identification. For open-search scenarios characterized by numerous unexpected modifications, however, such conventional approaches lead to a combinatorial search space explosion. Methods such as MODa (Na et al. 2012), Open-pFind (Chi et al. 2018), and MSFragger-LOS (Yu et al. 2020) have been developed to meet this challenge. By extracting tags from spectra for extension or employing a dual-indexing strategy that simultaneously scores both unmodified and modification-carrying fragment ions, these tools enable efficient, unbiased discovery of unknown modifications, substantially expanding the analytical depth of modification-centric proteomics.

To secure high-confidence results and improve identification sensitivity, a rescoring step can be applied after database searching to drive the evaluation metric from a single score to multi-dimensional statistical learning. For instance, PeptideProphet (Keller et al. 2002) employs a probability model to characterize the feature distributions for correct versus incorrect matches, whereas Percolator (Käll et al. 2007) utilizes a support vector machine (SVM) to learn an optimal decision boundary between them, effectively distinguishing true from false positives. Rescoring has become a vital component of the standard workflow, laying a more robust qualitative foundation for downstream analysis.

However, with the continuous innovation of mass spectrometry technology and the growing demand for the analysis of various complex samples, protein search engines are facing increasingly severe challenges. On one hand, spectra generated by new high-sensitivity instruments (e.g. Orbitrap Astral) are characterized by extremely high peak density and a wider dynamic range, posing new adaptability requirements for traditional search algorithms. On the other hand, the inherent analytical difficulties of several highly representative complex samples persist. For example, human leukocyte antigen (HLA) features high polymorphism and non-specific cleavage, presenting great difficulties for identification; metaproteomics faces enormous search spaces from diverse species; and single-cell data suffer from low peptide concentrations and weak signals, demanding high discriminatory power. Collectively, these challenges highlight the urgent need for next-generation, more powerful and intelligent search solutions.

Meanwhile, deep learning-based prediction tools have advanced rapidly, with models such as DeepRT (Ma et al. 2018), DeepLC (Bouwmeester et al. 2021), pDeep (Zhou et al. 2017, Zeng et al. 2019, Tarn and Zeng 2021), Prosit (Picciani et al. 2024), and AlphaPeptDeep (Zeng et al. 2022) demonstrating remarkable power in predicting peptide properties including retention time, fragment ion intensity, and ion mobility. Tools like MSBooster (Yang et al. 2023) (integrated into MSFragger) and Oktoberfest (Picciani et al. 2024) have further substantiated the utility of this information in rescoring stage, showcasing their potent effect on enhancing identification sensitivity.

Currently, a unified solution that simultaneously supports both restricted and open searches, integrates deep learning features, and maintains high sensitivity across diverse scenarios remains lacking. Although existing tools cover both restricted and open modes and even support data-driven rescoring, they still exhibit limited sensitivity on certain datasets, show suboptimal performance in open-search scenarios, and lack support for deep learning-enhanced rescoring of open search results. Here, we present pFind+, an iterative version of the pFind framework designed specifically for DDA workflows, featuring substantial redesigns of the raw scoring and rescoring pipelines as well as unifying restricted and open searches with integrated deep learning features. Our main contributions are as follows:

- We introduce a high-precision raw scoring module with an acceleration strategy for improved candidate filtering, coupled with evolved rescoring approaches that synergistically enhance sensitivity.
- We integrate deep learning features into rescoring with hardware-specific speedup, achieving significant identification gains. Notably, our framework supports deep learning-enhanced rescoring specifically for open-search results, enabling deeper PTM discovery.
- Comprehensive evaluations on diverse datasets demonstrate that our method achieves the highest sensitivity across all search modes, while maintaining robust quality control performance.

## Materials and methods

### Overview of the pFind+ architecture

**Figure 1:**
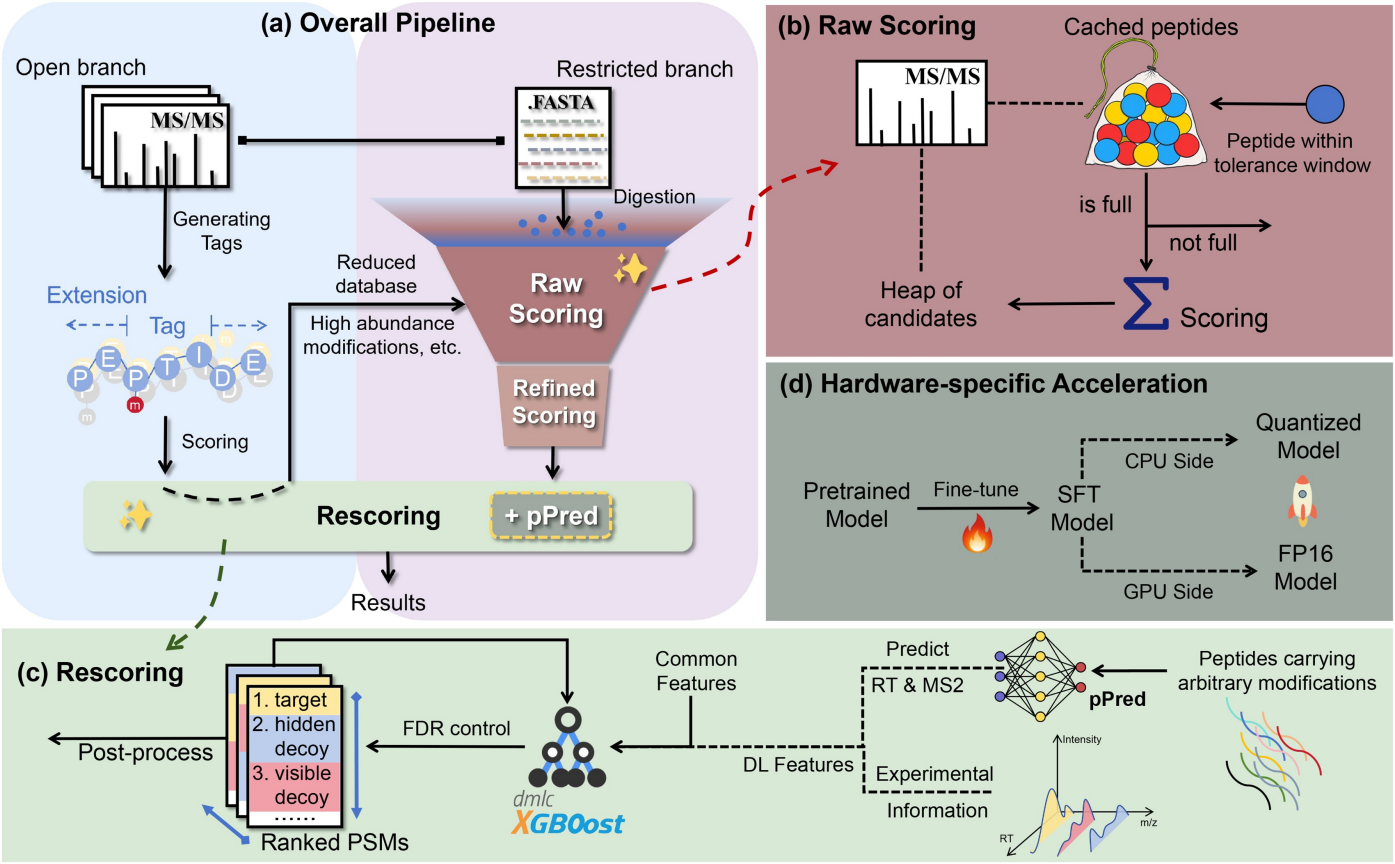
Overview of the pFind+ workflow. (a) Overall pipeline. (b-c) Implementation details of the raw scoring and rescoring modules. (d) Hardware-specific acceleration for pPred inference.

As depicted in Fig. 1a, pFind+ builds upon the dual-branch architecture originally introduced by Open-pFind. The open branch (Open-pFind+) employs tag indexing to match peptides with arbitrary modifications and learns optimal search parameters, which are subsequently used to shrink the database and fed into the restricted branch. Operating either independently or after the open branch, the restricted search begins with a raw scoring step to filter candidates, proceeds to refined scoring, and culminates in the rescoring stage for final verification.

In this framework, pFind+ introduces substantial redesigns at two of these critical stages: raw scoring and rescoring. For the former, we implemented a high-precision scorer that improves early candidate selection through exact fragment matching, with a specially architected acceleration framework. At the rescoring stage, candidate peptide-spectrum matches (PSMs) are iteratively reranked by a XGBoost-based model under an adapted error control mechanism. To further boost discrimination, deep learning-predicted peptide properties are used to derive auxiliary features; additionally, hardware-aware acceleration can be applied to ensure efficient inference. Together, these innovations enable more sensitive and reliable identification in DDA proteomics.

### High-precision raw scoring with acceleration

The raw scoring module performs an initial fragment-level matching between theoretical peptides and experimental MS/MS spectra, eliminating unlikely PSMs and retaining a limited set of candidates for subsequent stages. At this early step, both matching accuracy and computational efficiency are critical. The coarse-grained vectorization strategy employed by pFind and Comet, however, can produce substantial matching errors, compromising the effective retention of correct peptides. In this section, we present an improved raw scoring scheme that substantially enhances matching accuracy, while also maintaining competitive speed through a tailored acceleration strategy (Fig. 1b).

### Accelerated fragment matching with hash indexing

pFind+ implements a high-precision raw scoring that performs per-fragment matching at user-specified tolerances, eliminating the quantization errors inherent to coarse-grained vectorized methods. For each spectrum, experimental peaks are stored as a sorted array of floating-point m/z values. To accelerate the per-fragment search, we construct a linear hash index H mapping each integer bin I=⌊m/z⌋ to the starting position of peaks within that bin. For a theoretical fragment mass $m_{\text{theo}}$, we retrieve its bin in O(1) time and sequentially scan to locate peaks satisfying $|m/z-m_{\text{theo}}|<\text{tolerance}$. This hash-indexed search strategy reduces the average per-fragment lookup complexity from O(log n) to O(w) compared to binary search, where *n* is the total number of peaks and *w* is the average number of peaks per integer bin (typically w≪n).

While this hash-indexed method significantly improves precision and is algorithmically optimized, profile-guided analysis revealed that it incurs a substantial slowdown relative to the original vectorized method due to increased cache-unfriendly memory access overhead. In this regime, memory latency dominates computational cost, which is unacceptable for large-scale searches. We therefore implemented two other synergistic acceleration strategies, as detailed below, that effectively offset this performance penalty, allowing high-precision candidate selection to scale across large proteomic datasets.

### Cache-efficient batched peptide scoring

Rather than scoring each peptide immediately upon matching to a spectrum, we accumulate peptides in per-spectrum caches, with capacity *B* set to balance cache efficiency against memory footprint. When a spectrum’s cache reaches capacity, we execute a batch scoring pass that processes all cached peptides against its peak list sequentially, exploiting temporal locality to reduce cache misses. This reorders the computation from peptide-driven to spectrum-driven, amortizing memory access costs across the entire batch.

Following each batch pass, the peptides stored in cache are filtered into a min-heap data structure that maintains only the highest-scoring candidates up to a predetermined capacity. While a heap remains below capacity, new peptides are inserted directly; once full, each incoming peptide compares against the current minimum, replacing it when superior and triggering a heapify operation with logarithmic time complexity. This systematic processing prepares the cache for subsequent peptide batches. Algorithm 1 summarizes our scoring procedure.

Algorithm 1 High-Precision Raw Scoring with Acceleration

1: **Input:** Peptide *p*

2: **Global data:** Spectrum set S, per-spectrum caches C and min-heaps H, cache capacity *B*, heap capacity *M*

3: Identify candidate spectra $S_{p}\subseteq S$ (by m/z)

4: **for** each spectrum $s\in S_{p}$**do** 

5:  Append *p* to $C_{s}$ (via memory pool allocation)

6:  **if**$|C_{s}|=B$**then**▹ Cache full, trigger batch scoring

7:   **for** each cached peptide $p^{\prime }\in C_{s}$**do** 

8:    Compute theoretical fragment masses of $p^{\prime }$

9:    Compute *score* via hash-indexed matching

10:    **if**$|H_{s}|<M$**then** 

11:     

$$H_{s}.\text{push}(p^{\prime })$$

12:    **else if**$\mathrm{score}>H_{s}.\text{min}()$**then** 

13:     Replace heap root with $p^{\prime }$

14:     

$$H_{s}.\text{heapify}()$$

15:    **end if** 

16:   **end for** 

17:   Clear $C_{s}$ (via memory pool deallocation)

18:  **end if** 

19: **end for** 

### Low-overhead memory management via pool

The peptide caching strategy introduces a secondary performance bottleneck: the overhead of dynamic memory allocation. Each peptide object accounts for a variable memory footprint, depending on its sequence length, modifications, and associated metadata. As peptides are repeatedly inserted into and flushed from per-spectrum caches, these operations trigger frequent memory allocation and deallocation by the operating system (OS), an operation that is prohibitively expensive. Under high-throughput conditions, this overhead accumulates rapidly and dominates execution time, ultimately offsetting the performance gains achieved through the caching strategy and rendering the approach slower than the baseline hash-indexed method.

To eliminate this cost, we implemented a custom memory pool allocator. The pool pre-allocates a large, contiguous block of memory (expandable on demand to accommodate dataset scale), which is divided into uniformly sized chunks for management. When a new peptide object must be created, the allocator does not request memory from the OS. Instead, it rapidly assigns a pre-reserved chunk from the pool. Correspondingly, when a peptide object is no longer needed and the cache is flushed, its memory chunk is not returned to the OS. It is simply marked as free and returned to a thread-local free list, where it is immediately available for reuse by subsequent objects.

This design completely bypasses the OS’s general-purpose memory management, enabling extremely fast peptide object creation and clearance. A key feature is that each thread maintains its own local pool, eliminating resource contention and synchronization delays during multi-thread execution. Consequently, this memory pool sustains the high-frequency peptide cache updates required by the batched scoring strategy, preserving its computational advantages and ensuring the overall efficiency of the high-precision matching pipeline.

### Deep learning-augmented rescoring framework

Distinguishing true PSMs from high-scoring false positives requires powerful discrimination at the decision stage. In this section, we enhance the rescoring module with a high-performance classifier, along with a calibrated error control mechanism. Since conventional approaches rely primarily on hand-crafted features that are often insufficient to capture the underlying physicochemical consistency between peptides and spectra, we further incorporate deep learning-derived information into this stage, and ensure efficient inference through hardware-aware optimization (Fig. 1c). Our design is detailed below.

### XGBoost rescoring with calibrated FDR control

Conventional rescoring frameworks employ linear SVM-based classifiers, whose decision boundaries inadequately model the non-linear feature interactions inherent in PSMs, and require explicit kernel engineering to extend their capacity. SVM is well-suited for directional numeric features but struggles with categorical encodings whose numerical order carries no semantic meaning. We therefore adopted XGBoost, a gradient-boosting ensemble that naturally partitions heterogeneous features via split-point decisions and iteratively constructs decision trees to capture complex relationships, providing superior fitting capacity. This framework extracts richer information from the same data, enabling more sensitive PSM discrimination.

However, this direct substitution disrupts the statistical exchangeability between target and decoy PSMs. XGBoost’s enhanced expressivity allows it to exploit subtle decoy-specific patterns during training, which biases the model to favor false positives among high-confidence targets while suppressing decoys that would otherwise indicate these errors, thus leading to underestimation of the False Discovery Rate (FDR). Inspired by Freestone et al. (2025), we devised a reliability-calibrated protocol that partitions decoy PSMs into visible and hidden cohorts. Visible decoys serve as explicit negative training examples, while hidden decoys function as internal error surrogates for unbiased FDR estimation. In this way, we prevent the model from learning decoy-specific biases, guaranteeing reliable FDR estimates. Specifically:

- **Initialization:** Prior to rescoring, decoy PSMs are alternately assigned to hidden or visible categories, with identical peptide sequences forced into the same cohort to ensure balanced yet mutually exclusive partitions.
- **Training data selection:** Positive examples are high-confidence PSMs, which include a small fraction of hidden decoys meeting the same criterion. Negative examples are sampled from visible decoys.
- **Unbiased FDR estimation:** Following each rescoring iteration, spectra are sorted by the score of their best candidate in descending order, and at each rank *i* we compute:
  (1) $${\text{FDR}}_{i}=\frac{r\times \#\text{Hidden}\;\text{Decoys}\;\text{among}\;\text{top}\;i}{\#\text{Targets}\;\text{among}\;\text{top}\;i}$$

The factor *r* serves as a calibration term that accounts for the target-decoy ratio and the partitioning scheme of decoys. In the common setting where a 1:1 target-decoy ratio is assumed and decoys are equally divided into two cohorts, r=2 yields an unbiased estimate of the FDR.

This protocol exploits XGBoost’s discriminative power while rigorously controlling FDR, enabling high-sensitivity identification without statistical inflation.

### Data-driven feature augmentation via transfer learning

To further enhance the discriminative power of the rescoring module, we incorporated a peptide property prediction model to derive auxiliary features. Specifically, we employed pPred (Zhou et al. 2022), a deep learning-based predictor that pretrained on large-scale proteomic datasets—capable of predicting both retention time (RT) and MS/MS fragment intensities for peptides with arbitrary modifications. Unlike many existing tools limited to a fixed set of common modifications, pPred supports generic representation and prediction of over 1000 PTM types, including rare or user-defined ones, making it well-suited for open-search scenarios where unexpected or rare modifications are prevalent.

In practice, experimental variations in chromatographic systems and mass spectrometry parameters can introduce systematic biases relative to the pretrained model. We address this through lightweight fine-tuning on high-confidence PSMs from the target dataset (overfitting safeguards detailed in Note S, available as supplementary data at *Bioinformatics* online), which aligns the model with specific experimental conditions. Feature construction integrates these predictions as quantitative agreement metrics: the MS2 feature is computed as cosine similarity between observed and predicted fragment intensity vectors, i.e.


(2) $$\text{MS}2\_\text{feature}=\text{cos}(I_{\;\text{exp}\;},I_{\text{pred}}),$$

while the RT feature captures chromatographic consistency through absolute prediction error,


(3) $$\text{RT}\_{\text{feature}=|\text{RT}}_{\;\text{exp}\;}{-\text{RT}}_{\text{pred}}|$$

The symmetric error distribution of a well-calibrated RT predictor makes absolute deviation an effective measure of RT agreement. This deep learning-enhanced rescoring (hereinafter referred to as DL mode) is optionally enabled by the user.

### Trust-aware adaptation for open search

Open search often yields peptides carrying diverse modifications, some of which bear rare modifications that challenge reliable prediction. To maintain robust performance, we implement a dual strategy. First, peptides bearing extremely low abundance modifications are excluded from fine-tuning data to prevent unreliable patterns from corrupting model adaptation. Second, for modifications absent from the fine-tuning set, we introduce a trust-aware indicator that flags reduced prediction reliability to the rescoring model. This mechanism automatically down-weights candidates with uncertain predictions, preserving sensitivity for well-represented modifications while mitigating potential degradation from low-confidence cases. A comprehensive discussion distinguishing our method from existing approaches is provided in Note 2, available as supplementary data at *Bioinformatics* online.

### Hardware-coordinated acceleration for inference

Deep learning-based prediction introduces non-trivial computational overhead, especially in CPU-only environments where inference can become prohibitively time-consuming. To ensure practical feasibility and scalability, we implemented a suite of acceleration techniques that collectively enable substantial speedup.

- **Dataset-Level Deduplication**: Identical peptide forms (sequence + modifications + charge) frequently recur across spectra. We perform global deduplication prior to inference, reusing predictions to eliminate redundant computations.
- **Top-K Pruning** (*Optional*): We observed that the vast majority of confidently identified peptides originate from the high-ranking candidates of previous stages. By selectively processing only the top-3 or even top-1 candidates per spectrum, we eliminate the bulk of prediction workload with negligible impact on identification sensitivity.
- **Hardware-Specific Acceleration** (*Optional*): To leverage the underlying hardware capabilities, we deploy distinct strategies for CPU and GPU scenarios (Fig. 1d).
- For CPU-only environments, we apply dynamic quantization. It converts model weights from 32-bit floating-point to 8-bit integer (INT8) and dynamically quantizes activations at runtime, accelerating compute-intensive matrix operations through efficient integer arithmetic. This can achieve a nearly 3× speedup with minimal impact on prediction accuracy.
- On CUDA-enabled systems, we employ half-precision (FP16) computation, which doubles theoretical memory bandwidth and computational throughput. This yields approximately 1.5x acceleration with almost no compromise in sensitivity.

### Datasets and benchmark tools

#### Datasets

We evaluated pFind+ on six publicly available datasets covering diverse proteomics scenarios and instruments, including HLA (Chong et al. 2020), MetaProteome (Gavin et al. 2018), SingleCell (Guo et al. 2022), HEK293 (Chick et al. 2015), HeLa (Guzman et al. 2024), and the Nine-Species dataset (Tran et al. 2017) (Table 1, available as supplementary data at *Bioinformatics* online).

**Table 1:** Peptide identifications of different ablation configurations.

Configuration	HeLa	HLA
pFind	34141	16420
pFind+raw*	36234 (6.1%↑)	17441 (6.2%↑)
pFind+rescore*	38315 (12.2%↑)	18916 (15.2%↑)
pFind+	42619 (24.8%↑)	20688 (26.0%↑)

#### Benchmark tools

pFind+ was benchmarked against four widely used search engines: pFind v3.2.2, MSFragger v4.3 (FragPipe suite v23.1), MaxQuant v2.7.0.0, and Comet v2025.02.0. All datasets were searched using dataset-recommended parameters (see Table 2, available as supplementary data at *Bioinformatics* online). To ensure a fair comparison, search configurations were maximally harmonized across different software tools where feasible.

## Results

### Comprehensive performance evaluation of pFind+

To comprehensively compare different search strategies, we evaluated multiple search configurations spanning restricted and open search, with and without DL integration across five representative datasets at 1% peptide-level FDR. Specifically, restricted search was performed on all engines; open search was evaluated for tools supporting this mode (MSFragger-LOS, Open-pFind and Open-pFind+); DL-assisted restricted search was assessed on MSFragger+MSBooster and pFind+ with DL enabled; and deep learning-enhanced open search was evaluated on pFind+ with both open search and DL enabled. Of note, on the MetaProteome dataset, MaxQuant was excluded due to prohibitive runtime, and MSFragger-LOS could not be executed because of its excessive memory demand.

**Figure 2:**
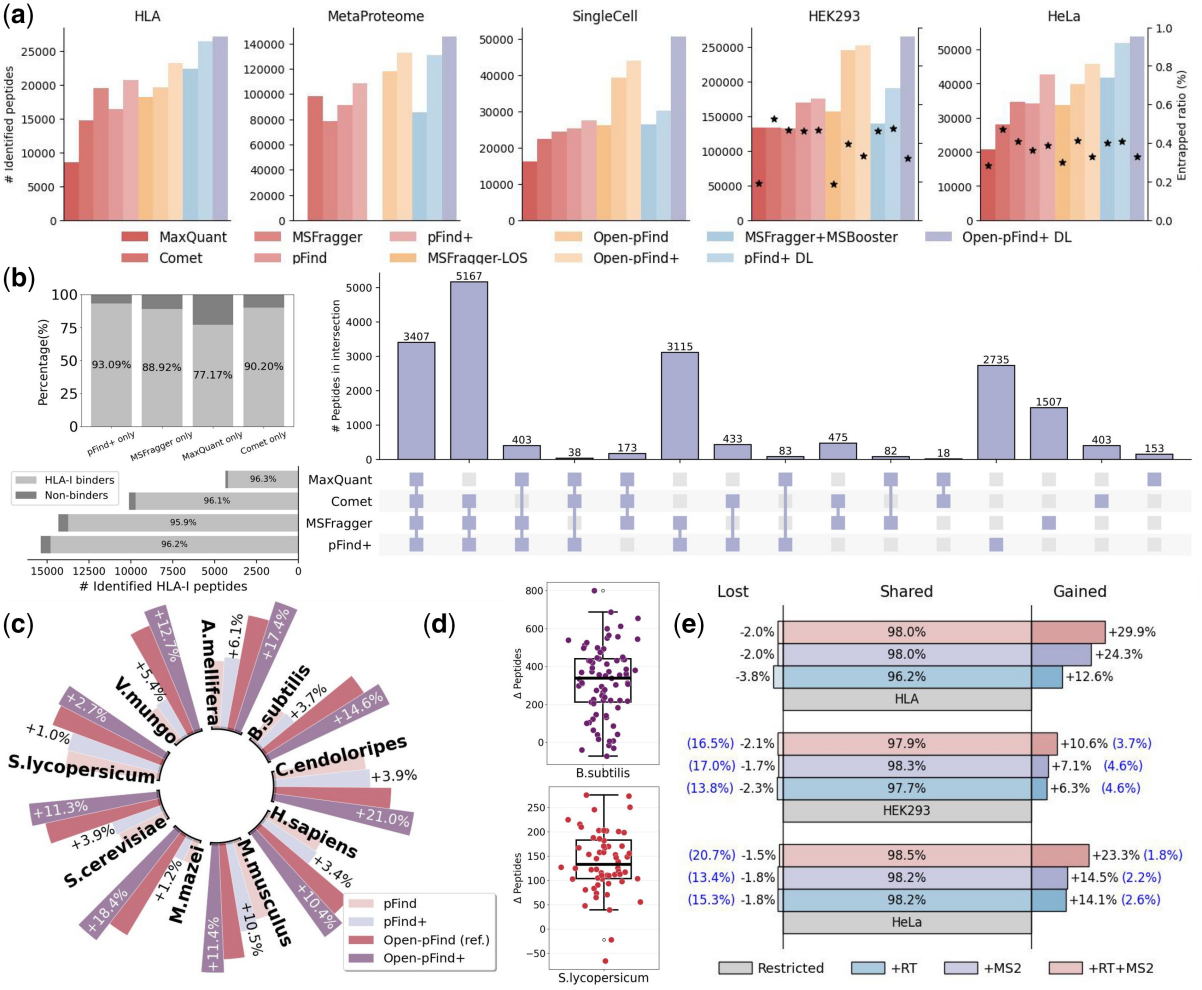
Comprehensive evaluation of pFind+. (a) Peptide identifications of 11 search workflows on five datasets, shown alongside entrapment ratios for HEK293 and HeLa. pFind+ consistently achieves the highest sensitivity across all datasets, with robust FDR control. (b) HLA-I peptide intersections across four engines. pFind+ identifies the most unique peptides while maintaining a low non-binder ratio (left panel). (c) Relative identifications on the Nine-species dataset. pFind+ surpasses pFind across all species in both restricted and open modes. (d) Per-run gains on *B. subtilis* and *S. lycopersicum*. pFind+ improves over pFind in most runs. (e) Deep learning features significantly boost sensitivity by recovering true signals and filtering false positives (entrapment ratios indicated in blue).

Across all datasets and modes, pFind+ consistently achieved the highest peptide identification sensitivity (Fig. 2a, Table 2, available as supplementary data at *Bioinformatics* online). In restricted search with DL mode off, pFind+ reports 3.8%–26.0% (average 16.0%) more peptides than pFind, and 5.7%–23.2% (average 14.7%) more than the best-performing alternative among the other three workflows. On HeLa data acquired from the new-generation Orbitrap Astral instrument, pFind+ identified 42,619 peptides, substantially surpassing all other engines. For open scenarios, Open-pFind+ identified 2.9%–18.5% (average 11.5%) more peptides than Open-pFind, and maintained a remarkable advantage over MSFragger-LOS across all datasets. Notably, while open search is generally expected to expand identification coverage, we observed that MSFragger-LOS exhibited degradation compared to its restricted baseline on certain datasets (and was even outperformed by pFind+ across all cases); whereas Open-pFind+ consistently showed stable improvements over its restricted counterpart (see Note 3, available as supplementary data at *Bioinformatics* online, for a detailed discussion). When incorporating DL features, the sensitivity of pFind+ was further enhanced. The addition of DL to our restricted search produced gains ranging from 8.5% on HEK293 to 27.9% on HLA (average 17.6%), which surpassed those achieved by MSFragger+MSBooster on every dataset, despite pFind+ starting from a higher baseline. Notably, pFind+ is the only tool that supports deep learning-enhanced open search, enabling comprehensive PTM discovery. By applying DL, it yielded an increase of 5.0%–17.3% (average 12.7%) in peptide identifications over Open-pFind+, and reached the highest identifications across all 11 search workflows. Further analysis of the uniquely identified peptides confirms that they did not exhibit systematic differences in charge state or peptide length compared to those identified by conventional methods (Note 4, available as supplementary data at *Bioinformatics* online). Collectively, our optimal configurations achieved gains of 12.7%–29.3% (average 17.9%) in restricted search and 8.0%–38.4% (average 25.8%) in open search over the best existing tools, establishing a substantial lead across both modes.

To validate the effectiveness of FDR control, we employed an entrapment strategy on the HeLa and HEK293 datasets by appending an Arabidopsis proteome to the search database (see Note 5, available as supplementary data at *Bioinformatics* online, for detailed methodology). Under a nominal 1% peptide-level FDR, rigorous error control corresponds to an expected entrapment ratio of approximately 0.44%, given the 5:4 target-to-entrapment composition of the database, with only minor stochastic fluctuations. pFind+ consistently maintained results within this reasonable range across all four modes evaluated. Moreover, the proportion of entrapped peptides remained stable after incorporating deep learning features, indicating that the enhanced discriminative power of the DL mode did not distort the target-entrapment balance.

We further evaluated the accuracy of HLA class I identification on the HLA dataset. MixMHCpred3.0 (Tadros et al. 2025) was used to predict HLA-I binding affinity (see Note 6, available as supplementary data at *Bioinformatics* online, for details), which provides an orthogonal assessment of the reliability of HLA identifications. Figure 2b shows the intersections across four engines, with pFind+ contributing the largest number of uniquely identified peptides. The left panel demonstrates that peptides identified exclusively by pFind+ contained the lowest proportion of non-binders. Importantly, pFind+ reported the largest number of peptides among all engines, but its overall non-binder ratio remained only slightly higher than that of MaxQuant, underscoring its strong performance on HLA data.

Given that the pFind series demonstrates superior performance on standard proteomics datasets, we conducted a focused comparison between pFind and pFind+ across a Nine-species dataset. Open-pFind+ consistently outperformed Open-pFind across all nine subsets, with improvements approaching 20% in several cases (Fig. 2c, Table 3, available as supplementary data at *Bioinformatics* online). By contrast, pFind+ yielded only modest gains over pFind, likely due to (1) the absence of certain high-abundance modifications from the recommended parameter set, and (2) diminishing returns as replicates approached saturation of the detectable peptide pool. Per-run analysis of the *B. subtilis* (77 runs) and *S. lycopersicum* (60 runs) datasets demonstrated consistent, incremental gains for pFind+ over pFind in nearly every run, yet the aggregate gain was far less than the sum of per-run improvements, supporting the saturation hypothesis (Fig. 2d).

To further dissect the impacts of incorporating distinct deep learning features, we systematically evaluated the effects of adding RT, MS2, or both to the restricted search workflow. The MS2 feature contributes more than the RT feature within the context of our model (Fig. 2e). Incorporating any of these features enabled the retention of the most peptides identified by the original workflow, while simultaneously capturing a substantial number of additional peptides. Of note, the small fraction of peptides lost exhibited a significantly higher entrapped ratio compared to the newly identified ones. The integration of both features led to further improvements beyond those achieved by either alone, underscoring the complementary nature of the two feature types.

### Evaluation of hardware-aware inference acceleration

**Figure 3:**
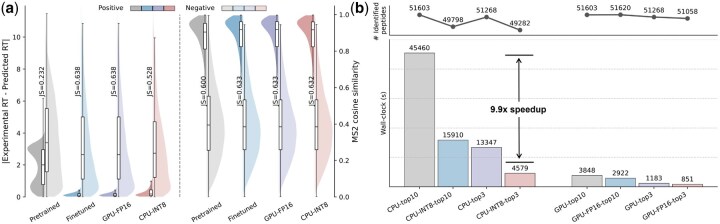
Performance of hardware-specific acceleration. (a) Discriminative power of RT and MS2 under different model variants. INT8 quantization slightly degrades RT but preserves MS2; FP16 on GPU maintains full fidelity. (b) Speed-sensitivity trade-off. Top-3 pruning with INT8 achieves a ∼10× speedup on CPU with 4.5% sensitivity loss, and Top-3 pruning with FP16 delivers a ∼4.5× speedup on GPU with minimal sensitivity loss.

In order to evaluate the acceleration efficacy of our DL mode, we began by comparing the performance of the supervised fine-tuned (SFT) model, the INT8-quantized model (CPU side), and the FP16 model (GPU side). Using the HeLa dataset as a representative benchmark, we fine-tuned the RT and MS2 models on high-confidence identifications (q-value ≤ 0.001) from the restricted search to obtain the SFT model. Thereafter we constructed a validation set comprising target peptides with q-value ≤ 0.01 (excluding those used for fine-tuning) as positive examples, and decoy peptides with q-value ≤ 0.5 as negatives. The discriminative power of features derived from each model variant was quantified using Jensen-Shannon divergence (Fig. 3a). Consistent with our previous discussion, FP16 inference on GPU could be employed without any practical compromise, achieving considerable acceleration while preserving model fidelity. For CPU, INT8 quantization has minimal impact on the MS2 model but moderately impairs the performance of the RT model. Additionally, the fine-tuning step proved essential for the RT model, without which it would exhibit poor discrimination between positive and negative samples. In contrast, the pretrained MS2 model in this case displayed remarkable robustness even without task-specific adaptation.

We further analyzed the trade-off between acceleration and identification sensitivity (Fig. 3b). As anticipated, CPU inference without optimization was computationally prohibitive, requiring approximately 12.5 hours. On both platforms, pruning candidate lists from the original top-10 to top-3 yielded a dramatic reduction in inference time with only a minor loss in sensitivity. On CPU, top-3 pruning alone yielded superior benefits compared to INT8 quantization, making it preferable to the latter when this level of speedup is sufficient. For users requiring extreme acceleration, we advocate combining top-K pruning with INT8 quantization, which achieved nearly 10-fold speedup (reducing runtime to 1.25 hours) at a modest sensitivity cost of 4.5%. On GPU, FP16 inference alone introduced no practical performance penalty. When coupled with top-3 pruning, it achieved a 4.5-fold acceleration, lowering inference time to approximately 14 minutes. In summary, we recommend enabling the DL mode on GPU-equipped systems for optimal performance, or adopting the aggressive CPU acceleration strategy when GPU resources are unavailable, thereby maintaining an advantageous balance between speed and sensitivity.

### Scoring module-level ablation study

To systematically evaluate the contributions of our raw scoring and rescoring modules, we performed ablation studies on two representative datasets: HeLa and HLA, which were specifically selected to represent scenarios of high spectral complexity and expansive search space, respectively. We compared four configurations in restricted search mode: (i) pFind, (ii) pFind+, and two intermediate variants—(iii) pFind enhanced only with the high-precision raw scoring (pFind+raw*), and (iv) pFind enhanced only with the improved rescoring (pFind+rescore*).

As shown in Table 1, applying the enhanced rescoring alone substantially increased peptide identifications, yielding gains of approximately 12.2% on HeLa and 15.2% on HLA over the baseline. By comparison, employing the high-precision raw scoring alone provided relatively modest improvements of 6.1% and 6.2%. This is expected because raw scoring primarily serves as a high-throughput front-end filter for an enormous search space, and its benefit cannot be fully realized without sufficiently powerful downstream discrimination. When paired with pFind’s legacy SVM-based rescoring, the promising candidates rescued by the enhanced pre-filter cannot be consistently promoted to top-ranking under a stringent FDR threshold, thereby limiting the observable gain. This interpretation is supported by directly comparing pFind+rescore* with the complete pFind+ system—the addition of high-precision raw scoring to the already improved rescoring pipeline accounts for the remaining 12.6% and 10.8% sensitivity gains, bridging the gap between pFind and pFind+. Notably, when both modules are jointly optimized, their combined improvement exceeds the sum of their individual contributions, indicating not only a super-additive synergy but also demonstrating the mutual indispensability of the two modules.

### Efficiency evaluation of raw scoring optimizations

**Figure 4:**
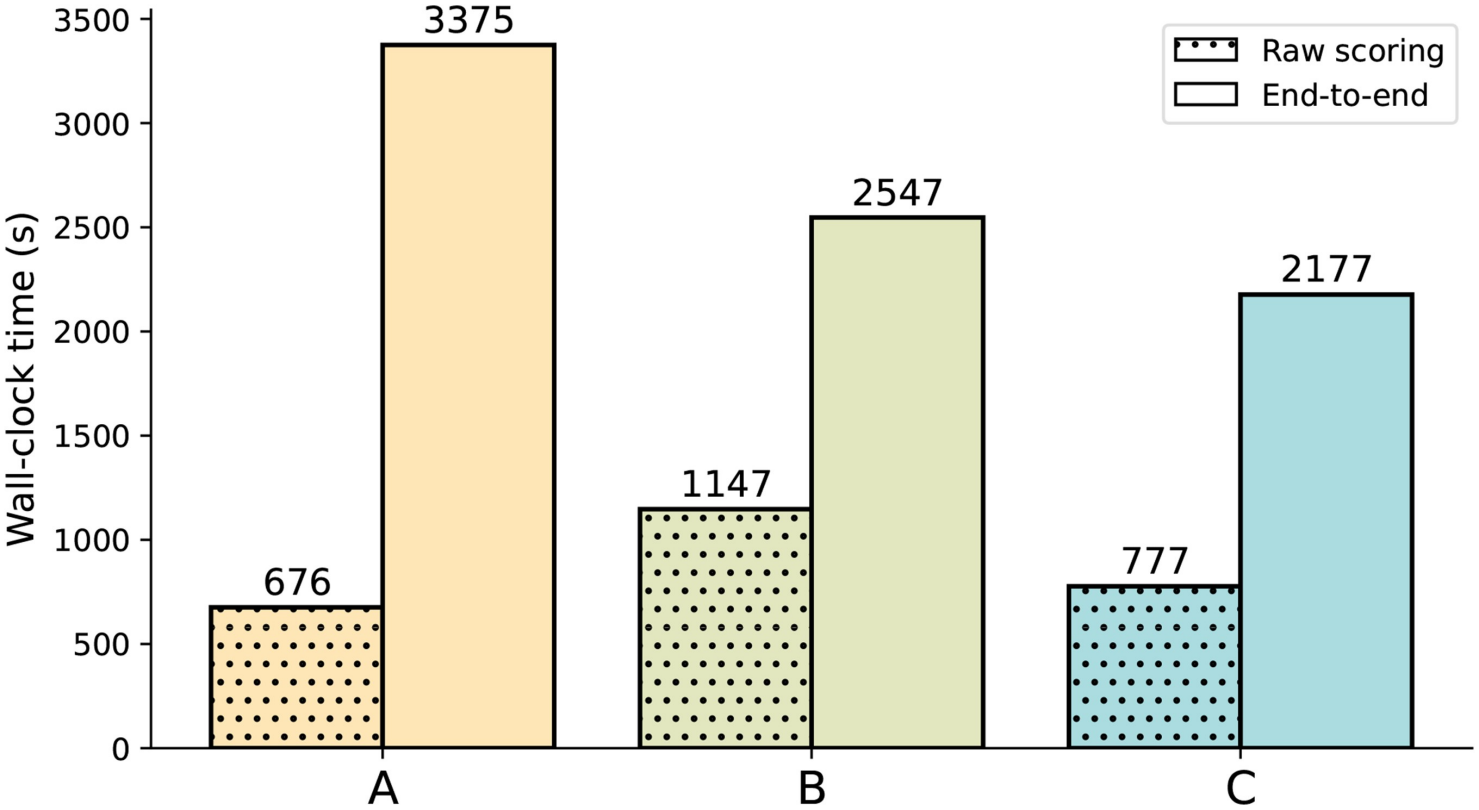
Runtime comparison of different raw scoring implementations. Our method (C) is slightly slower than the vectorized baseline (A) but significantly faster than the hash-indexed method (B).

To quantify the efficiency of our proposed high-precision raw scoring mechanism, we benchmarked three scoring strategies on the HeLa dataset in terms of runtime: (A) the legacy integer-based vectorized scoring in pFind, (B) hash-indexed high-precision matching, and (C) our batched peptide caching with memory pool allocation methodology (Fig. 4).

Method A unsurprisingly achieved the highest speed, owing to its cache-contiguous memory access pattern enabled by vectorization and binary-level operations. In contrast, method B, though delivering substantially improved MS/MS peak-matching accuracy, incurred an approximately 1.7× slowdown compared to method A, noticeably reducing throughput. Method C, which is the raw-scoring strategy adopted in pFind+, further accelerates method B through more sophisticated techniques, recovering much of the lost efficiency. Although its runtime remains slightly higher than that of method A, the discrepancy is within an acceptable margin, especially given the significant gain in pre-screening efficiency. Moreover, pFind+ implements acceleration across the entire pipeline, including binary I/O of intermediate files and other algorithmic refinements. Therefore, when measuring the end-to-end runtime of restricted search, pFind+ achieves an overall speedup of approximately 1.55× relative to pFind. The enhanced sensitivity, together with the reduced runtime, makes pFind+ a more effective alternative to pFind.

### Modification-level analysis

Finally, we performed a modification-centric analysis on the HEK293 dataset to further examine the impact of deep learning in our open search workflow. This dataset was selected for its large identification yield and high prevalence of modified peptides, which can provide a robust foundation for this analysis. For each PTM observed in more than 500 distinct peptides—a threshold chosen to ensure that the expected number of entrapment peptides is at least 2 (based on an estimated entrapment ratio of 0.44%) for statistical reliability, we calculated the corresponding entrapment ratio under both the Open-pFind+ and Open-pFind+ DL.

**Figure 5:**
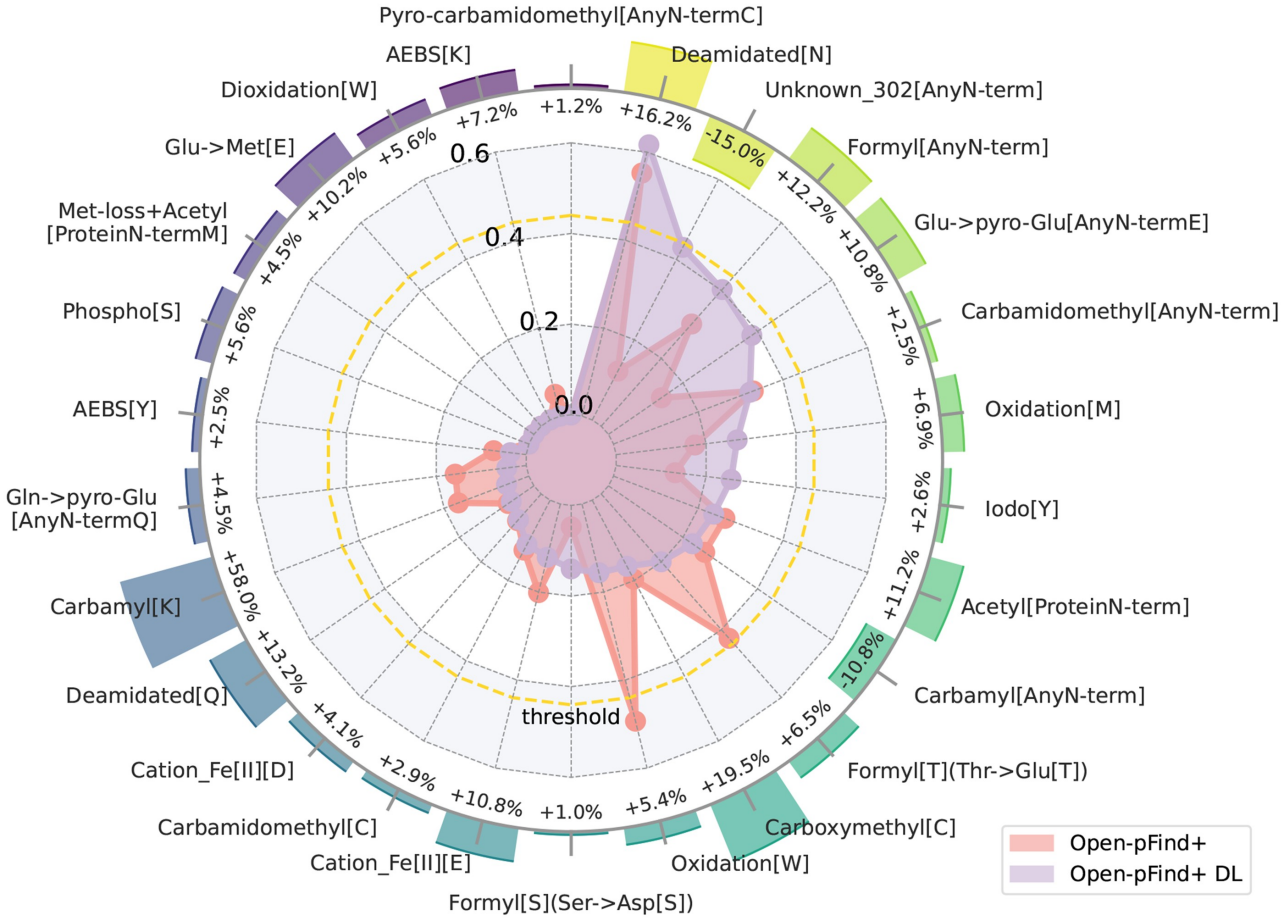
Modification-level entrapment ratios (inner radar) and DL gain ratios (outer bars) for high-frequency PTMs in HEK293. Most PTMs maintain baseline entrapment ratios in both modes.

As shown in Fig. 5, the entrapment ratios for the vast majority of PTMs in both modes cluster within the expected range established by our peptide-level baseline. Notable deviations between the two modes for certain modifications occur primarily because their carriers hover just above the 500-frequency threshold, so that minor fluctuations in the number of entrapped peptides are enough to swing the ratio. The Deamidation on asparagine (Deamidated[N]), despite its high occurrence frequency (more than 6500 instances) in both modes, exhibits the most elevated entrapment ratio. This indicates that the near-isobaric (∼1 Da) mass shift associated with this modification is intrinsically challenging and more prone to driving erroneous identifications. In addition, the integration of DL features yields a consistent increase in identification counts across most PTMs. A detailed quantitative characterization of the trust-aware mechanism, which underpins the reliability of this approach, is provided in Note 7, available as supplementary data at *Bioinformatics* online. Consequently, our DL-enhanced open search achieves significant gains in sensitivity without systematically distorting error rates at the modification level, thereby supporting robust PTM discovery with enhanced depth.

## Conclusion and discussion

Protein search engines are critical for translating mass spectrometry data into biological insights. pFind+ distinguishes itself as a leading solution. Its novel high-precision raw scoring and XGBoost-based rescoring modules work synergistically, achieving superior identification sensitivity in both restricted and open modes while maintaining robust statistical control and competitive speed. Furthermore, with the incorporation of deep learning, pFind+ reaches the highest sensitivity across all benchmarks and uniquely enables DL-enhanced open search, providing unprecedented depth in PTM discovery.

Future work will focus on developing a Linux version to expand platform compatibility. We will also explore integration with data-independent acquisition (DIA) workflows to extend applicability. Additionally, efforts will be made to continue enhancing its predictive models that adopt more complex yet effective techniques such as graph neural networks. In summary, pFind+ provides a powerful, integrated solution for deeper and more confident proteomic discovery.

## Supplementary Material

btag224_Supplementary_Data

## Data Availability

The data used in this study are available from ProteomeXchange (https://www.proteomexchange.org/), with details provided in Table 1, available as supplementary data at *Bioinformatics* online. The pFind+ software is implemented in C++ for Windows and is freely available at https://pfnd.ict.ac.cn/se/pFind_plus.
