# Supplementary material for: Advancing proteomic discovery through optimized multi-stage scoring and deep learning-enhanced open search
Source: Bioinformatics. 2026 Jul 7;42(Suppl 1):btag224. doi: 10.1093/bioinformatics/btag224 (PMC13340261; doi:10.1093/bioinformatics/btag224)
Supplement: btag224_Supplementary_Data [file btag224_supplementary_data.pdf]

Supplementary Tables

Table S1. Evaluation datasets.

Dataset	Description
HLA	HLA class I and II peptides from tumor and matched normal tissues of cancer patient C3N02671 (PXD013649), selected as a representative case from this well-established benchmark cohort.
MetaProteome	Host and microbial proteins from stool samples of subjects at risk for type 1 diabetes (PXD008870).
SingleCell	Human GV-stage oocyte proteins from oocyte maturation studies (PXD023366).
HEK293	Human HEK293 cell lysates acquired on the Q Exactive mass spectrometer (PXD001468).
HeLa	HeLa cell lysates acquired on the Orbitrap Astral mass spectrometer (PXD046453).
Nine-species	Datasets spanning 9 species: *Vigna mungo* (PXD005025), *Mus musculus* (PXD004948), *Methanosarcina mazei* (PXD004325), *Bacillus subtilis* (PXD004565), *Candidatus endoloripes* (PXD004536), *Solanum lycopersicum* (PXD004947), *Saccharomyces cerevisiae* (PXD003868), *Apis mellifera* (PXD004467) and *Homo sapiens* (PXD004424).

Footnotes: Evaluations were conducted on six publicly available datasets. 1) Five datasets were used to benchmark pFind+ against four other search engines, encompassing 11 distinct workflows. 2) The Nine-species dataset was used for a direct comparison between pFind+ and pFind in both restricted and open search modes.

Table S2. Search parameters across all datasets.

	HeLa	HEK293	HLA	MetaProteome	SingleCell	Nine-species
Precursor tolerance	10ppm	20ppm	20ppm	20ppm	20ppm	20ppm
Fragment tolerance	10ppm	20ppm	0.02Da	20ppm	20ppm	20ppm
Fixed modifications	C(+57)	C(+57)	/	C(+57)	C(+57)	C(+57)
Variable modifications	nAc, M(+16)	nAc, M(+16)	M(+16)	M(+16), N(+1)	nAc, M(+16)	nAc, M(+16)
Peptide length range	6-100	6-100	8-25	7-50	6-100	7-63
Enzyme type	trypsin	trypsin	/	trypsin	trypsin	trypsin
Enzymatic cleavage specificity	specific	specific	non-specific	specific	specific	specific
Maximum missed cleavage	2	3	2	2	3	2

Abbreviations: C(+57) refers to the carbamidomethylation of cysteine (C); M(+16) refers to the oxidation of methionine (M); N(+1) refers to the deamidation of asparagine (N); nAc refers to the N-terminal acetylation of proteins.

Decoy Generation: Decoy sequences for all search engines were generated by reversing the corresponding target sequences at the start of the search process and employed throughout the workflow, following standard practice in proteomics.

Notes:

The search parameters listed above were specified by the original data producers, or adopted from established field standards where explicit recommendations were unavailable. To ensure a fair comparison, these configurations were maximally harmonized across all search engines. Specific exceptions were made only when dictated by software constraints: for MaxQuant, which lacks a maximum peptide length parameter (using maximum mass instead), we approximated the length limit by setting the maximum mass to max_length $\times$ 100 Da (assuming an average residue mass of 100 Da); for Comet, which does not support ppm-based fragment tolerance, we uniformly applied a fixed tolerance of 0.02 Da. Regarding peak filtering, parameters varied significantly across search engines due to differing tool capabilities. Generally, the number of peaks was constrained between 10 and 200, with the exception of the HeLa dataset, which allowed a broader range of 10 to 1,000. Specifically, pFind imposes only a maximum peak limit, whereas Comet and MaxQuant enforce only a minimum peak limit.

Table S3. Number of peptide identifications by different search workflows across five datasets at 1% peptide-level FDR.

Workflow	HLA	MetaProteome	SingleCell	HEK293	HeLa
MaxQuant	8584	/	16105	133451	20814
Comet	14806	98482	22467	133875	27956
MSFragger	19576	92427	24331	145558	34601
pFind	16420	91184	26051	169048	34141
pFind+	**20688**	**108735**	**27622**	**175501**	**42619**
MSFragger-LOS	18265	/	26109	156953	33735
Open-pFind	19622	117995	40644	244994	39899
Open-pFind+	**23256**	**132917**	**44068**	**251993**	**45862**
MSFragger+MSBooster	24470	101202	26344	153406	41712
pFind+ DL	**26467**	**130844**	**30232**	**190493**	**51915**
Open-pFind+ DL	**27156**	**145474**	**50635**	**264664**	**53776**

Footnotes: 1) The numbers in bold denote the best results. 2) Since MaxQuant does not support peptide-level FDR control, it applies FDR control at PSM level, which is less stringent and can indirectly inflate reported peptide counts. 3) On the MetaProteome dataset, MaxQuant was excluded due to prohibitive runtime, and MSFragger's open search could not be executed because of its excessive memory demand.

Table S4. Number of peptide identifications by pFind, pFind+, Open-pFind and Open-pFind+ on Nine-species dataset at 1% peptide-level FDR.

	Restricted Search		Open Search	
Species	pFind	pFind+	Open-pFind	Open-pFind+
Vigna mungo	22688	23910 (5.4%↑)	65351	73623 (12.7%↑)
Mus musculus	8851	9781 (10.5%↑)	12577	14006 (11.4%↑)
Methanosarcina mazei	29589	29951 (1.2%↑)	145053	171729 (18.4%↑)
Bacillus subtilis	61315	63604 (3.7%↑)	172773	198052 (14.6%↑)
Candidatus endoloripes	14523	15086 (3.9%↑)	18381	22250 (21.0%↑)
Solanum lycopersicum	87389	88267 (1.0%↑)	108799	111735 (2.7%↑)
Saccharomyces cerevisiae	42177	43837 (3.9%↑)	97714	108762 (11.3%↑)
Apis mellifera	36869	39107 (6.1%↑)	63502	74549 (17.4%↑)
Homo sapiens	26502	27410 (3.4%↑)	51058	56352 (10.4%↑)

Supplementary Notes

S1 Overfitting Safeguards of the Fine-tuning Procedure

To prevent overfitting, the following safeguards were implemented during fine-tuning:

- Validation split: The training data were randomly split into 90% for training and 10% for validation, providing an independent set for monitoring generalization.
- Rollback mechanism: The evaluation metrics (MSE for RT, Smooth L1 for MS2) were monitored on the validation set throughout fine-tuning. To prevent transient fluctuations from prematurely triggering early stopping, a rollback was performed whenever validation metrics worsened compared to the previous epoch, after which the checkpoint of the preceding epoch was reloaded and the current epoch was retrained. Up to three rollbacks were allowed per training session.
- Early stopping: Training was halted when the validation metrics failed to improve by at least 0.1% (relative) for three consecutive epochs.
- Epoch limit: A maximum of 30 epochs was set as a hard upper bound.

The Adam optimizer was used with an initial learning rate of 0.001, decayed by a factor of 0.95 after each epoch to a minimum of 5e-5.

S2 Comparison of Transfer Learning Strategies

To clearly differentiate our transfer learning strategy from existing methods, we systematically compared the adaptation mechanisms employed by representative tools in the field.

MS2Rescore employs a static transfer approach: it directly applies pre-trained models (MS2PIP, DeepLC) without any online adaptation mechanism. Consequently, it relies entirely on the model's inherent generalization, failing to correct for experiment-specific deviations or handle unknown modifications unseen during training.

INFERYS utilizes a Prosit-derived model but restricts its transfer learning to hyperparameter optimization (automatic collision energy selection) rather than parameter-level adaptation. It performs no weight updates to align with the target data distribution. Moreover, its pre-trained knowledge is strictly limited to unmodified peptides and methionine oxidation, rendering the transfer fundamentally inapplicable to the diverse modification space required for open search.

Oktoberfest, built on Prosit, implements a partial transfer strategy: it fine-tunes the RT model weights to adapt to chromatographic shifts but relies on collision energy interpolation (without weight adaptation) for MS2 intensity transfer. Crucially, like the aforementioned tools, its transferred knowledge is confined to known modifications, preventing effective adaptation to the novel chemical patterns encountered in open-search tasks.

MSBooster, leveraging external models such as DIA-NN or Prosit, adopts a hybrid transfer approach: it uses non-linear regression (LOESS) to calibrate RT and ion mobility predictions but applies no parameter adaptation for MS2 intensities. For unknown modifications, it attempts

a heuristic transfer via rigid ”peak-shifting” (copying unmodified patterns), which fails when modifications alter fragmentation physics. Furthermore, its inability to transfer RT/IM knowledge to unknown modifications precludes its use in open-search scenarios.

Our method distinguishes itself in three aspects. First, our source model is pre-trained on open-search results, encoding predictive patterns for over a thousand modification types to ensure broad initial coverage. Second, we employ parameter-level fine-tuning for both RT and MS2 models, enabling non-linear adaptation of the learned weights to the specific experimental setup, moving beyond simple linear corrections. Third, a trust-aware mechanism is introduced to mitigate the impact of unreliable modifications inherent to open-search data.

S3 Discussion on MSFragger-LOS Degradation in Specific Datasets

The performance degradation of MSFragger-LOS observed in certain datasets stems from its strategy of employing an extremely wide mass window to capture peptides with unknown modifications. This approach drastically expands the search space, intensifying competition for spectrum matches compared to restricted searches; consequently, some peptides correctly identified by the baseline MSFragger may be outcompeted and replaced by coincidental high-scoring matches attributed to unknown modifications, a risk that is particularly pronounced in datasets with low modification abundance where false positives are more likely to arise. Furthermore, unlike the pFind+ framework which employs a remedial two-step strategy of database reduction followed by a refined restricted search, MSFragger-LOS operates as a single-pass process without such a sensitivity recovery mechanism. This structural difference leads to a dependency on sample characteristics: while MSFragger-LOS successfully identifies more peptides than the baseline in PTM-abundant datasets (e.g., HEK293 and SingleCell, where Open-pFind+ also showed substantial gains), it exhibits reduced identification numbers in samples with lower modification burdens, where the cost of the expanded search space outweighs the benefits of discovering sparse modifications.

S4 Validation of DL-Enhanced Identifications: Property Distributions of Uniquely Identified Peptides

We systematically compared the distributions of charge state, peptide length, and PSM scores for peptides uniquely identified by pFind+ DL against those found by established methods (pFind, MSFragger, MaxQuant, and Comet) on HEK293 dataset. Regarding PSM scores, since scoring systems vary across engines and are not directly comparable, we specifically compared the DL mode against pFind (using scores derived from the refined scoring module). We also performed similar comparisons in the open-search scenario. All results are provided in Supplementary Figures 1 and 2.

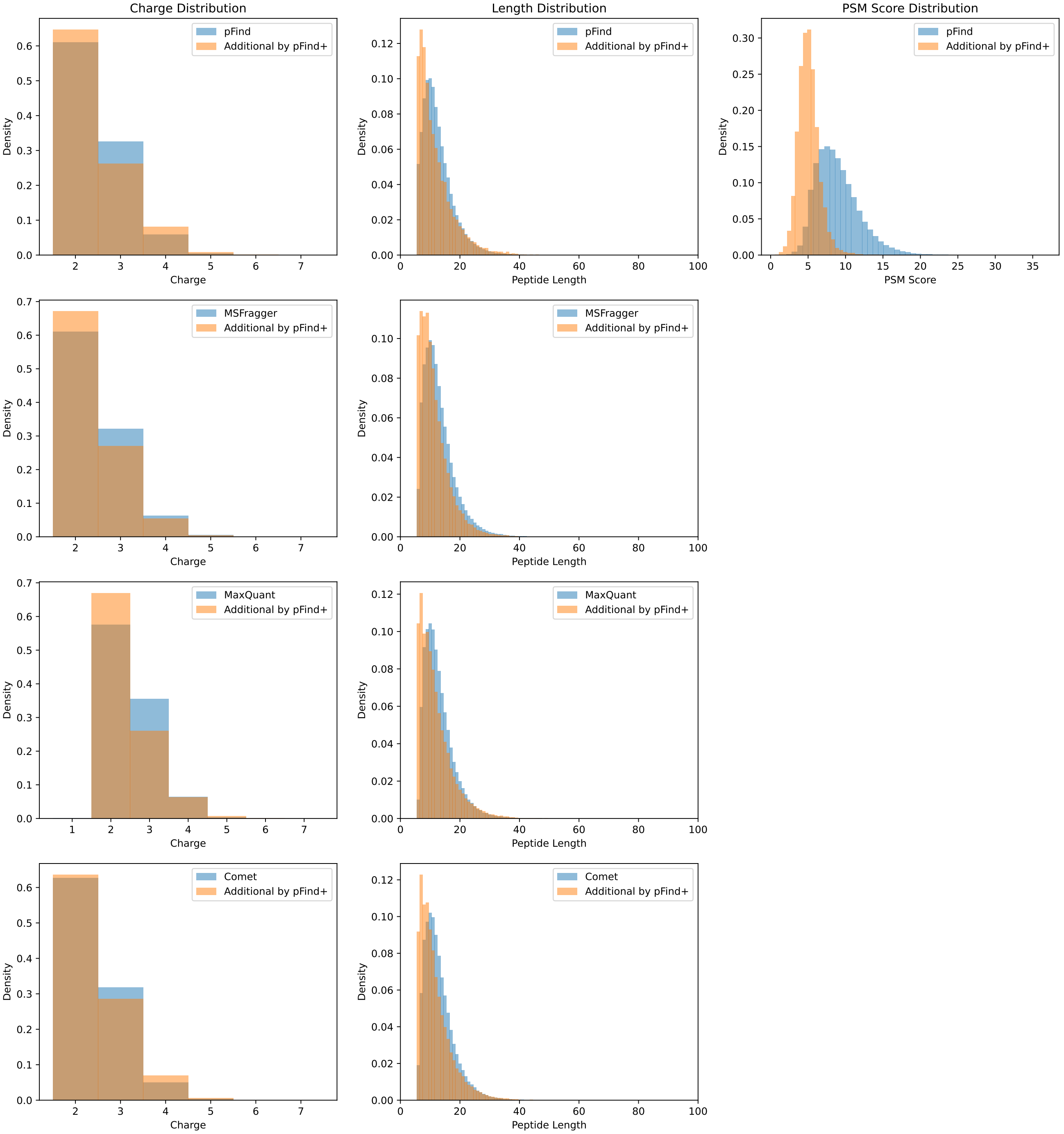

Supplementary Figure 1. Comparison of charge state, peptide length (and PSM score) distributions between peptides uniquely identified by DL mode and those identified by other established methods.

As observed, peptides uniquely identified by pFind+ DL (and Open-pFind+ DL) do not differ systematically in charge state or peptide length compared to conventional identifications. However, we do observe differences in their PSM score distributions. This is expected, as the DL-enhanced mode is specifically designed to rescue peptides that may be less prominent in conventional scoring metrics. If these uniquely identified peptides were indistinguishable from those found by conventional methods across all properties, there would be no reason for them to have been

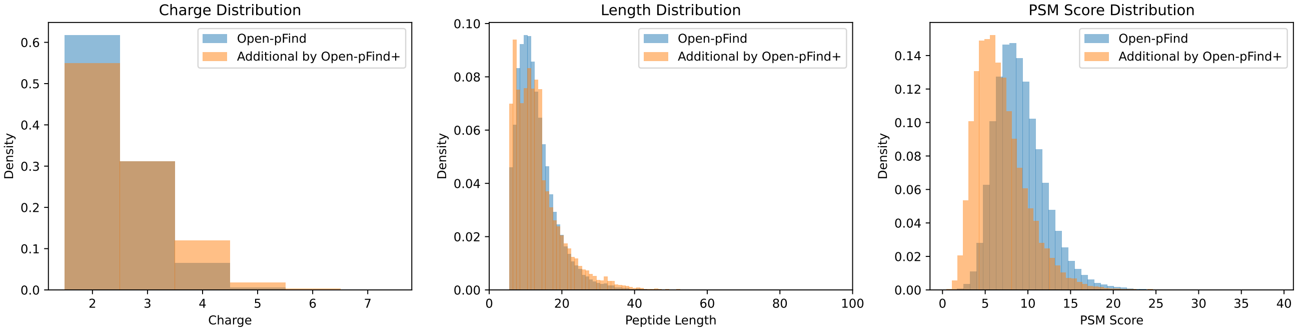

Supplementary Figure 2. Comparison of charge state, peptide length (and PSM score) distributions between peptides uniquely identified by Open-pFind+ DL and those identified by other Open-pFind.

missed by traditional methods in the first place. Specifically, the peptides uniquely identified by DL mode show a slight shift toward lower PSM scores. This precisely demonstrates the value of the deep learning approach: it rescues peptides that may have suboptimal traditional scores but exhibit higher consistency with their true fragmentation patterns and retention times as captured by deep learning features.

S5 FDR Filtering Details in Entrapment Searches

The entrapment searches were applied and evaluated at the peptide-level FDR, consistent with the primary analytical workflow. For each distinct peptide identified under 1% FDR threshold, we tracked its origin (target, decoy, or entrapment) and counted each unique peptide only once. The entrapment ratio was then calculated as the number of entrapment peptides divided by the number of target peptides. Given the proportion of the entrapment database relative to the entire search space, any false identifications in the target set would be expected to distribute proportionally between entrapment and true sequences; therefore, the observed entrapment ratio provides a direct assessment of FDR control quality.

S6 Validation of HLA Class I Identifications Using MixMHCpred3.0

To assess the reliability of HLA class I peptide identifications, we performed binding affinity prediction using MixMHCpred3.0 following the original study's patient-specific alleles. The HLA alleles used were: A01:01, A02:01, B08:01, B44:02, C05:01, C07:01. The tool was executed with the following command:

```
./MixMHCpred -i seqs.txt -o output.txt -a A0101,A0201,B0801,B4402,C0501,C0701
```

by which MixMHCpred3.0 reports, for each peptide sequence, the best %Rank among all alleles, and a %Rank$\leq$2 is considered a potential binder according to its publication.

S7 Quantitative Reporting on the Trust-Aware Mechanism

We first quantified the frequency of activation of this mechanism by analyzing the proportion of peptides with "unreliable modification" predictions among all candidates participating in rescoring (spectrum_num × top10). Our statistics show that such peptides account for $1122626/11949862 \approx 9.4\%$ in the HeLa dataset, representing a substantial portion. Without targeted handling, a fraction of these unreliable peptides could be erroneously assigned high scores due to spurious advantages in deep learning features.

In implementation, the trust-aware mechanism introduces an additional binary feature (f14, the 15th feature with 0-based indexing) to identify whether a peptide's deep learning features are compromised by an unreliable modification prediction (where 1 denotes reliable and 0 denotes unreliable). During rescoring, the XGBoost model automatically learns the significance of this indicator. To visually demonstrate how this feature influences ranking, we extracted the trained XGBoost model from the best-performing rescoring run on the HeLa dataset. Supplementary Figure 3 presents three subtrees containing f14 (from the 2nd, 7th, and 9th trees, respectively) within the ensemble.

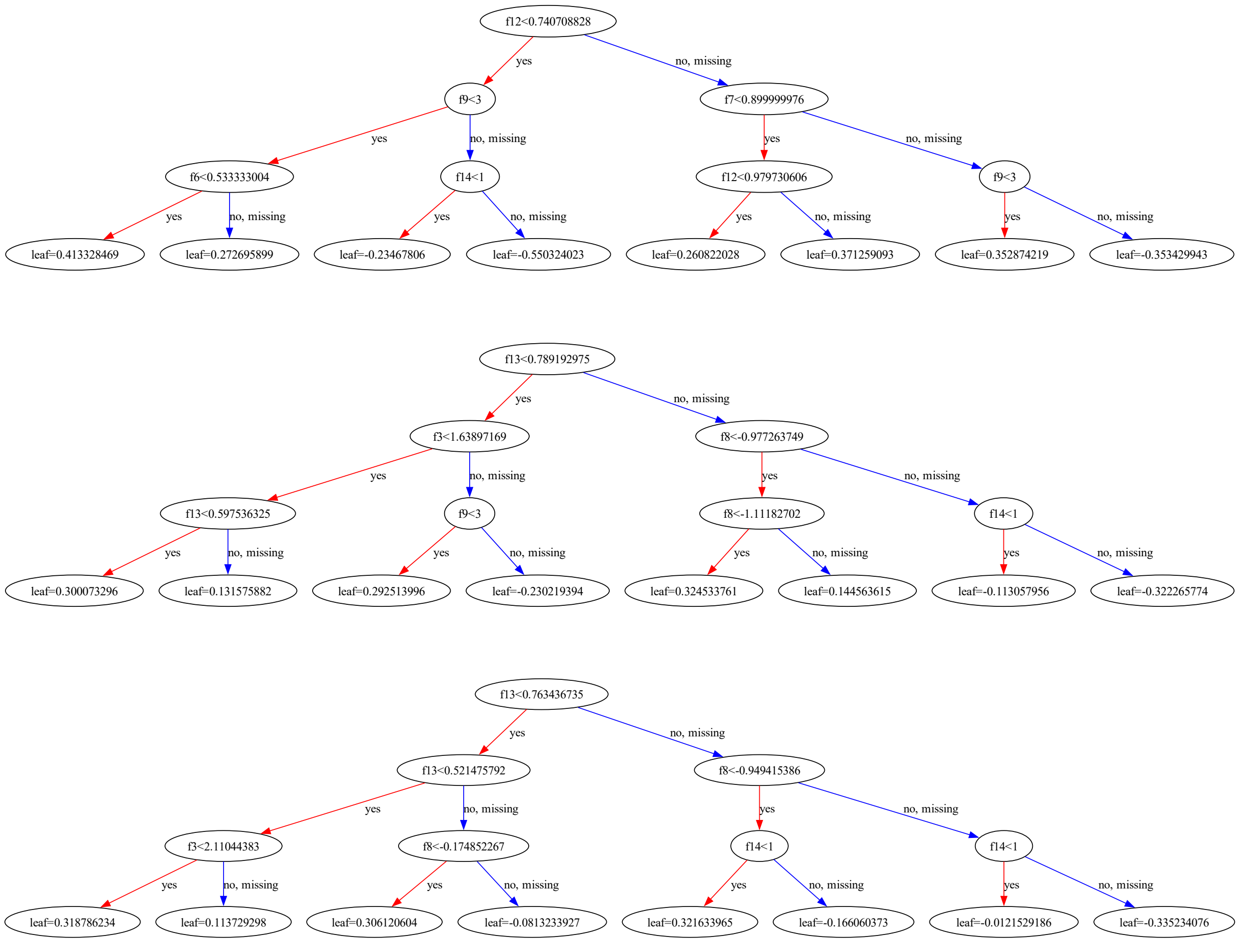

Supplementary Figure 3. Examples of subtrees containing the trust-aware indicator f14.

As observed, f14 frequently appears as a low-level splitting node, forming decision paths alongside f12 (RT feature) and f13 (MS2 feature). Given that target and decoy labels were assigned 0 and 1, respectively, during training, a smaller leaf node value corresponds to higher confidence (i.e., a stronger tendency towards target). The visualization reveals that when $f14 = 1$, samples are directed to branches with lower leaf values compared to when $f14 = 0$ (<1, where the indicator flags the prediction as unreliable). This demonstrates that XGBoost automatically learns to leverage this indicator to appropriately down-weight peptides when deep learning features pose reliability risks, thereby effectively controlling false positives introduced by unreliable modifications without compromising sensitivity for common modifications.

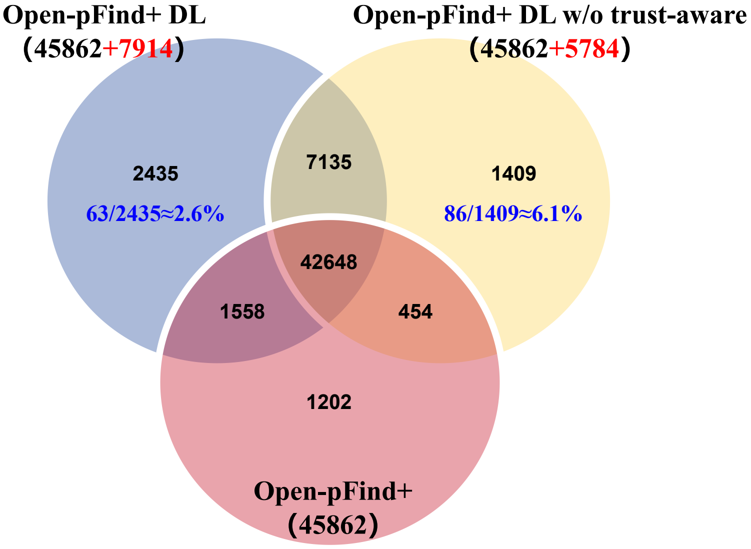

Supplementary Figure 4. Venn diagram of peptide identifications across three workflows on HeLa. Blue numbers denote the entrapment ratio for uniquely identified peptides.

Finally, we performed a control experiment without the trust-aware mechanism (denoted as Open-pFind+ DL w/o trust-aware). The Supplementary Figure 4 shows that, compared to the version without this mechanism, incorporating the trust-aware component yields additional relative gains of approximately 36.8%, on top of the substantial improvements already achieved by Open-pFind+ DL w/o trust-aware. Additionally, we compared the entrapment ratios of peptides uniquely identified by the two settings, and found that the ratio without the trust-aware mechanism was more than twice that with it. Together, these results confirm that the trust-aware mechanism boosts sensitivity while maintaining rigorous FDR control.
